# Principles and innovative technologies for decrypting noncoding RNAs: from discovery and functional prediction to clinical application

**DOI:** 10.1186/s13045-020-00945-8

**Published:** 2020-08-10

**Authors:** Yu-Meng Sun, Yue-Qin Chen

**Affiliations:** MOE Key Laboratory of Gene Function and Regulation, State Key Laboratory for Biocontrol, School of Life Sciences, Sun Yat-sen University, Guangzhou, 510275 People’s Republic of China

**Keywords:** Novel ncRNAs, Sequencing technologies, Functional ncRNA discovery, Subcellular localization, ncRNA database, Diagnostic kits

## Abstract

Noncoding RNAs (ncRNAs) are a large segment of the transcriptome that do not have apparent protein-coding roles, but they have been verified to play important roles in diverse biological processes, including disease pathogenesis. With the development of innovative technologies, an increasing number of novel ncRNAs have been uncovered; information about their prominent tissue-specific expression patterns, various interaction networks, and subcellular locations will undoubtedly enhance our understanding of their potential functions. Here, we summarized the principles and innovative methods for identifications of novel ncRNAs that have potential functional roles in cancer biology. Moreover, this review also provides alternative ncRNA databases based on high-throughput sequencing or experimental validation, and it briefly describes the current strategy for the clinical translation of cancer-associated ncRNAs to be used in diagnosis.

## Background

More than half a century after being considered as the central component in the central dogma of biology, RNA has been accepted to play various essential roles in different biological processes [1–4]. With recent developments in sequencing methods and information analysis, an increasing number of novel ncRNAs have been identified, including long noncoding RNAs (lncRNAs) [5, 6], circular RNAs (circRNAs) [7, 8], and novel small ncRNAs [9–11]. Growing studies have uncovered the characteristics of these ncRNAs, including their origins, mechanisms of generation, structures, and potential functions [6, 8, 12], which can be summarized into a principle for the identification of known species of ncRNAs or even novel ncRNA discovery. As many ncRNAs exhibit highly tissue-specific expression patterns and important roles in biological processes related to cancer [13–19], ncRNAs have been considered as ideal therapeutic targets for cancer diagnosis and treatment [20–22]. Due to the enormous transcription potential of mammalian genomes and multiple mechanisms of ncRNA generation [8, 9, 23, 24], the ncRNA world is still full of infinite mysteries, in which unknown species of RNAs could play important roles. Technological innovation makes it possible to discover more novel functional ncRNAs.

This review focuses on the principles and innovative technologies currently available for the discovery of novel ncRNAs or functional ncRNAs within specific subcellular compartments. The particular classes of ncRNAs that are either novel transcripts or “old dogs” performing “new tricks” are especially emphasized. Moreover, this review also provides an overview of ncRNA-associated databases and applications of cancer-related ncRNA identification for therapeutic strategies.

### Principle for novel ncRNA discovery

Early sequencing data revealed that the mammalian genome encodes many thousands of noncoding transcripts, especially those that resemble message RNAs (mRNAs) in length and splicing structure but cannot code for proteins, revealing that the world of RNA genes is far more complex than originally imagined [25]. Here, we summarized the features into a principle that could be used for the identification of known species of ncRNAs or even for novel ncRNA discovery.

### Chromatin signatures for novel ncRNA discovery

**Fig. 1:**
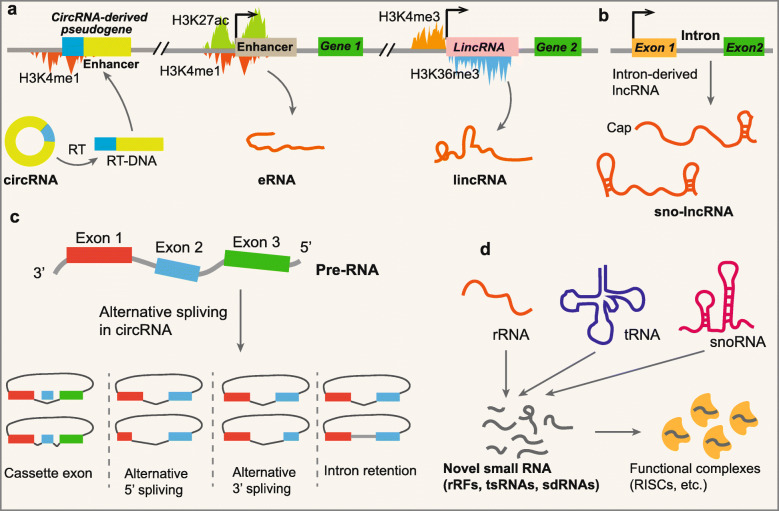
Principle for novel ncRNA discovery. **a** Identification and classification of ncRNAs based on chromatin signatures. Most mRNA-like lincRNAs are generated from genomic regions with H3K4me3/H3K36me3 signatures; eRNAs originate from activated enhancers with H3K4me1/H3K27ac signatures; the junction site sequences of circSTATB1 were reverse transcribed and inserted into an enhancer with active H3K4me1 signatures. **b** Sno-lncRNAs maintain their stability by their classical stem-loop structures of snoRNAs. **c** Alternative splicing within circRNAs. **d** A number of novel small ncRNAs derived from rRNAs (rRFs), tRNAs (tsRNAs), and snoRNAs (sdRNAs) have also been found to be enriched in RNA-induced silencing complexes (RISCs) and function in a miRNA-like pathway

The definition of genes has become a major hurdle following the sequencing of the human genome. As histones can be modified in different ways that are indicative of the underlying DNA functional state [26–29], chromatin modifications of the corresponding genomic region could represent important biological information for the identification and classification of noncoding transcripts. The increased occurrence of trimethylation of lysine 4 of histone 3 (H3K4me3) at the promoter regions of transcripts and trimethylation of lysine 36 of histone 3 (H3K36me3) along the entire transcribed region is a signature for active transcription; these occurrences are always found at active sites of mRNA transcription [27, 28]. By searching for H3K4me3/H3K36me3 signatures that failed to overlap with known genes, there was the identification of approximately 2500 regions in the human genome and approximately 1600 regions in the mouse genome that were actively transcribed [30, 31]. However, the vast majority of these intergenic regions with H3K4me3/H3K36me3 signatures produced multi-exonic RNAs that had a little capability to encode a conserved protein; they were termed as long intergenic ncRNAs (lincRNAs) (Fig. 1a) [30, 32]. A fraction of genes encoding ncRNAs display monomethylation of lysine 4 of histone 3 (H3K4m1) and histone H3 acetylation at lysine 27 (H3K27ac), which cover their initiation sites, indicating that they are transcribed from activated enhancers as enhancer-derived RNAs (eRNAs) (Fig. 1a) [29, 33]. Although both lincRNAs and eRNAs are categorized as lncRNAs because of their lengths, distinguishing different classes of ncRNAs based on distinct chromatin modifications is necessary because specific ncRNAs generated from given gene regulatory elements could function in classic modes [34, 35]. For example, eRNAs are thought to play an important role in regulating the 3D architecture of chromosomes near their site of transcription [34].

With developments in sequencing technologies and bioinformatics analysis, novel ncRNAs generated from alternative splicing processing or degradation of their parent RNAs have been discovered [8, 9, 36]. This kind of ncRNA does not have independent genomic regions or transcriptional regulatory elements and can be produced following parent gene transcription or degradation. Therefore, it is unable to accurately identify and describe the characteristics of these kinds of ncRNAs at the level of chromatin modification. As a typical example, circRNAs are mainly generated from alternative splicing of precursor RNA (pre-RNA), and then, they form covalently closed loop structures [8, 37]. Exonic circRNAs are produced from back-spliced exons of precursor linear RNAs, including mRNAs and lncRNAs, and they account for a major portion of the circRNA family. In addition, the intron lariats escaping from degradation can also form intronic circRNAs. Although there are some other variant forms of circRNAs, such as circular formats of small nucleolar RNAs (snoRNAs) and P RNA [38], the majority of circRNAs in humans are mainly produced from actively transcribed mRNA and lncRNA genes with H3K4me3-H3K36me3 signatures [39, 40]. Interestingly, the junction site sequences of circRNAs, such as circSTATB1 in mice, have been discovered to be inserted into an enhancer with active H3K4me1 signatures (Fig. 1a) [41]. The H3K4me1 modifications suggest that the functions of circRNAs in the regulation of enhancer and genome structure by forming pseudogenes, which may provide evidence for further classification of circSTATB1 as a retrotransposed circRNA (Fig. 1a) [41]. Although chromatin modifications cannot be used in the discovery of circRNAs, the modification signatures may be useful for more detailed classification of circRNAs.

In addition to circRNAs, there are many other novel ncRNAs that are generated from the degradation of typical transcripts from well-known genomic regions [9, 11, 42]. The excised intron-derived lncRNAs with snoRNA-like ends (sno-lncRNAs) are formed when one intron contains two snoRNA genes [42]. After splicing, the sequences between two snoRNAs escape degradation, resulting in the accumulation of certain lncRNAs. Another example is novel functional small ncRNAs, such as small ribosomal RNA-derived fragments (rRFs) [11], tRNA-derived small RNAs (tsRNAs) [9], and snoRNA-derived RNAs (sdRNAs) [10], which are derived from “old dogs” including ribosomal RNAs (rRNAs), transfer RNAs (tRNAs), and snoRNAs. An increasing number of discoveries of novel ncRNAs have indicated the limitation of chromatin modification signatures in novel ncRNA identification. However, chromatin signatures are still an available tool of ncRNA classification for efficient investigation of their functions.

### Principles for evaluating coding potential

As ncRNAs, especially lncRNAs and circRNAs, are likely to contain open reading frames (ORFs) purely by chance, it has been a challenge to determine whether a transcript is noncoding [43]. As a growing number of studies have shown that several lncRNAs and circRNAs can produce functional micropeptides [44–47], it is necessary to evaluate the RNA coding potential of novel ncRNAs.

The lack of evolutionary conservation in identified ORFs is evidence for the absence of coding potential of ncRNAs [48, 49]. Novikova et al. reported that a human lncRNA, SRA, has different isoforms that either function at the ncRNA level or produce proteins, and there is higher evolutionary stabilization of the RNA structural core than that of the translational product under evolutionary pressure [50]. Another example is Xist, a lncRNA involved in X chromosome inactivation in mammals that originates from the protein-coding gene *Lnx3* [51]. Interestingly, the *Lnx3* gene is still a protein-coding gene in opossum; however, it has been transformed into a noncoding transcript with frame-shifting mutations in later vertebrates [51]. In addition, the lack of homology to known protein domains and the inability to template significant protein production are the other important factors that are needed to be considered [48, 49]. These principles have been generalized to classify ncRNA coding potential by scoring conserved ORFs across diverse species with computational methods [52, 53], by searching for homology using protein-domain databases [54], and by sequencing ncRNAs associated with polyribosomes [55].

**Table 1:** Characteristics of diverse sequencing methods

Classification	Techniques	Short description	Strengths of the approach	Weakness	Ref
Microarrays	Tiling arrays	A method based on probes for discovering transcripts from specific genomic regions.	This approach can provide in-depth analysis of transcripts from target regions of genome.	Suffer from potential noise as a result of weak binding or cross-hybridization of transcripts to probes.	[56]
	Microarrays	A method based on a large number of oligonucleotide probes for performing quick global or parallel expression analysis of transcriptome.	Small size and high-throughput capabilities.	This method is not able to discover novel transcripts.	[57]
RNA-seq	RNA-seq	A technique that is currently the most widespread sequencing technology for both detecting RNA expression and discovering novel RNAs.	The method provides a global high-throughput detection amd identification of RNAs greater than 200 nt.	Its standard procedure is not suitable for detection of RNAs less than 200 nt. It also suffer from sequence errors at the reverse-transcription step or primer bias.	[58]
	RNA capture sequencing	A derivative technology combining RNA-seq with tilling arrays.	The method can specifically elevate the sequencing depth of target regions.	Suffer from disadvantages of both tiling arrays and RNA-seq.	[59]
scRNA-seq	Smart-seq	A scRNA-seq method based on a full-length cDNA amplification strategy.	Provide a full-length cDNA amplification of polyadenylated RNAs.	The limitations are lack of strand-specific identification, inability to read transcripts longer than 4 kb and only for polyadenylated RNAs.	[60]
	DP-seq	A scRNA-seq method using heptamer primers.	Suitable for smaller size samples or transcripts longer than 4 kb. this approach also suppresses highly expressed rRNAs in the cDNA library.	Captured RNAs are limited to polyadenylated RNAs.	[61]
	Quartz-seq	A scRNA-seq method which reduces back ground noise.	Reduce background noise by using specially suppression PCR primers to reduce side products.	The method is limited to detecting polyadenylated RNAs.	[62]
	SUPeR-seq	A single-cell universal polyadenylated tail-independent RNA sequencing.	Detect polyadenylated and nonpolyadenylated RNAs. Minimal rRNAs contamination.	Relatively low sensitivity for nonpolyadenylated RNAs.	[63]
	RamDA-seq	A full-length total RNA-sequencing method for analyzing single cells.	High sensitivity for nonpolyadenylated RNAs. It can also uncover the dynamics of recursive splicing.	Unknown	[64]
Small RNA-seq	Small RNA-seq	A type of RNA-seq that discriminate small RNA from larger RNA to better evaluate and discover novel small RNAs.	Specifically detect and discover small or intermediate-sized RNAs with target sizes.	Adapter ligation bias lead to reverse transcription bias or amplification bias.	[65]
Single-cell small-RNA sequencing	Small-seq	A method which detect small RNAs in a single cell.	The method can detect small RNAs in a single cell.	The limination may be similar to small RNA-seq.	[66]
Nascent RNA-seq	GRO-seq	A method labeling nascent RNAs with 5Br-UTP and immunoprecipitating RNAs for sequencing.	Detect nascent RNAs and provide a genome-wide view of the location, orientation, and density of Pol II-engaged transcripts.	The method is confounded by contamination due to nonspecific binding, which could possibly result in experimental bias.	[67]
	SLAM-seq	A method distinguishing nascent RNA from total RNA via s^4^U-to-C conversion induced by nucleophilic substitution chemistry.	It is an enrichment-free method which can avoid contamination induced by affinity purification.	The oxidation condition caused certain oxidative damage to guanine, which may impact the accurancy of sequencing.	[68]
	TimeLapse-seq	A method distinguishing nascent RNA from total RNA via s^4^U-to-C conversion induced by an oxidative nucleophilic aromatic substitution reaction.	It is an enrichment-free method which can avoid contamination induced by affinity purification.	The oxidation condition caused certain oxidative damage to guanine, which may impact the accurancy of sequencing.	[69]
	AMUC-seq	A method distinguishing nascent RNA from total RNA via transforming s^4^U into a cytidine derivative using acrylonitrile.	More efficient and reliable because it has a minimal influence on the base-pairing manner of other nucleosides.	Unknown	[70]
Identification of RNA-chromatin interaction	GRID-seq	A method that aims to comprehensively detect and determine the localization of all potential chromatin-interacting RNAs.	Use a bivalent linker to ligate RNA to DNA in situ and provide exact profiles of RNA-chromatin interactome.	Usable sequence length for mapping RNA is 18–23 bp. However, short sequence length can result in ambiguity in mapping.	[71]
	iMARGI	A method providing a in situ mapping of RNA-genome interactome.	iMARGI needs less number of input cells and is suitable for paired-end sequencing.	Unknown	[72]
	ChAR-seq	A chromatin-associated RNA sequencing that maps genome-wide RNA-to-DNA contacts.	Uncover chromosome-specific dosage compensation ncRNAs, and genome-wide trans-associated RNAs.	The method needs more than 100 million input cells.	[73]
Identification of RNA-RNA interaction	CLASH	A relatively early method that uses UV cross-linking to capture direct RNA-RNA hybridization.	Avoid noise from protein intermediate-mediated interactions.	This method only detects the RNA-RNA interactions base on proteins.	[74]
	RIPPLiT	A transcriptome-wide method for probing the 3D conformations of RNAs stably associated with defined proteins.	The method can capture 3D RNP structural information independent of base pairing.	This method only detects the RNA-RNA interactions base on proteins.	[75]
	MARIO	A method identifying RNA-RNA interactions in the vicinity of all RNA-binding proteins using a biotin-linked reagent.	This method can identify RNA-RNA interactions in the vicinity of all RNA-binding proteins.	The method only detects the RNA-RNA interactions base on proteins.	[76]
	PARIS	Psoralen analysis of RNA interactions and structures with high throughput and resolution.	Directly measure RNA-RNA interactions independent of proteins in living cells.	Unknown	[77]
	LIGR-seq	A method for the global-scale mapping RNA-RNA interactions in vivo.	Provide global-scale mapping RNA-RNA interactions independent of proteins in vivo	Unknown	[78]
	SPLASH	A method providing pairwise RNA-RNA partnering information genome-wide.	Map pairwise RNA interactions in vivo with high sensitivity and specificity, genome-wide.	Unknown	[79]
	RIC-seq	RNA in situ conformation sequencing technology for the global mapping of intra- and intermolecular RNA-RNA interactions.	The method performs RNA proximity ligation in situ and can facilitate the generation of 3D RNA interaction maps.	Unknown	[80]
	RNA proximity sequencing	A method based on massive-throughput RNA barcoding of particles in water-in-oil emulsion droplets.	This method can detect multiple RNAs in proximity to each other without ligation and is fit for studying the spatial organization of RNAs in the nucleus.	Unknown	[81]
RNAs in protein complexes or subcellular structures	FISSEQ	A method that offers in situ information of RNAs at high-throughput levels.	Provide information of RNAs at high-throughput levels. Visualization.	Unknown	[82]
	CeFra-seq	A method that physically isolates subcellular compartments and identifies their RNAs.	The methods have high sensitivity for low-abundance transcripts.	The method is limited to isolation protocols and the purity of resulting isolates.	[83]
	APEX-RIP	A method can map organelle-associated RNAs in living cells via proximity biotinylation combined with protein-RNA crosslinking.	The technique can offer high specificity and sensitivity in targeting the transcriptome of membrane-bound organelles.	Unknown	[84]

However, the coding potential of some novel ncRNAs, especially circRNAs, could fail to be determined with the principle mentioned above. Most circRNAs derived from mRNA back-splicing lose translational capacity because of the lack of effective ORFs or ribosome entry approaches, while a few circRNAs from coding or noncoding transcripts could also obtain novel ORFs and may be translated into new proteins [47, 85]. The deficiency of coding-potential evaluation could be due to the incomplete circRNAs databases across diverse species, the complex mechanism of ribosome entry and translational initiation of circRNAs [86], and the lack of databases that document the information of new peptides or proteins transcribed from novel templates containing the sequences of circRNA junction sites. Ribosome profiling has provided a strategy to identify ribosome occupancy on RNA, which has been proposed to be an available method for distinguishing noncoding transcripts from coding ones [55]. Nevertheless, some transcripts playing clear roles as ncRNAs have been detected in ribosomes, indicating that an association of RNA with a ribosome alone cannot be taken as evidence of protein-coding potential [87, 88]. These ribosome-associated ncRNAs may serve as translational regulators or may produce nonfunctional translation noise [89, 90]. Thus, experimental technologies such as mass spectrometry proteomics have been used to improve the accuracy of noncoding transcript definition [91].

### Characteristics of known ncRNAs

With the development of sequencing methods and information analysis, a vast number of diverse types of ncRNAs have been identified, such as microRNAs (miRNAs), lncRNAs, circRNAs, and novel small ncRNAs derived from well-known RNAs. Understanding the characteristics of the known ncRNAs would be helpful for novel ncRNA discovery.

NcRNAs are very heterogeneous in terms of their length and conformation [92]. They can be separated into 3 categories: (1) small ncRNAs (< 50 nt), including miRNAs (19–25 nt) [93], small interfering RNAs (siRNAs, 19–29 nt) [94], piwi-interacting RNAs (piRNAs, 25–31 nt) [95], and other functional small RNAs such as transcription initiation RNAs (tiRNAs, 17–18 nt) [96], tsRNAs (14–36 nt) [9], sdRNAs (17–24 nt or > 27 nt) [10], and sectional rRFs (15-81 nt) [11]; (2) intermediate-sized ncRNAs (50–500 nt), including 5S rRNAs (~120 nt) [97], 5.8S rRNA (~150 nt) [98], tRNAs (76–90 nt) [99], snoRNAs (60–300 nt) [100], and small nuclear RNAs (snRNAs, ~150 nt) [101]; (3) long noncoding transcripts greater than 500 nt, including linear lncRNAs [30] and circular circRNAs [40].

Most large ncRNAs, including lncRNAs and circRNAs, have been reported to be tissue-specific and expressed at relatively low levels [24, 102–104]. Different types of ncRNAs have distinct structures that maintain their stability. The most abundant lncRNAs are transcribed by RNA polymerase II (Pol II), and then, they undergo mRNA-like posttranscriptional processes, leading to 5′-caps and polyadenylated tails at their 3′ ends [30]. However, studies of novel ncRNA identification that were not based on polyadenylated tails have shown the existence of nonpolyadenylated ncRNAs such as sno-lncRNAs with snoRNA-like ends and circRNAs (Fig. 1b, c) [42]. Several sno-lncRNAs have been reported to stabilize their structures by interacting with classical snoRNA binding proteins (snoRBPs) via the classical stem-loop structures of snoRNAs (Fig. 1b) [105]. In addition, circRNAs are processed to form covalently closed loop structures without open terminals, which makes them resistant to degradation by exonucleases, causing them to have relatively high stability (Fig. 1c) [8]. In contrast, most eRNAs are nonpolyadenylated transcripts that have shorter half-lives than polyadenylated lncRNAs and are difficult to discover according to their even lower levels in organisms [24, 106].

Intermediate-sized and small ncRNAs possess specifically structural features as well, such as the conversed stem-box structures of snoRNAs (C/D box or H/ACA box) [100], unique 5′-caps of snRNAs (5′-trimethylguanosine caps or 5′-monomethylphosphate caps) [101, 107], the cloverleaf-like secondary structure of tRNA [99], and hairpin loop of miRNA precursor. Most types of intermediate-sized and small ncRNAs do not have specific modification at the 5′ or 3′ ends, and they maintain their stabilities via binding specific proteins to form complexes. For example, snoRNAs stabilize their structures by interacting with classical snoRBPs via the classical stem-loop structures [108]. Another example is miRNA, whose precursor yileds a miRNA:miRNA duplex with Dicer processing [109]. In most cases, only one strand of the deplex is usually incorporated into the RNA-induced silencing complex (RISC) to exist and function, and the other free strand is normally degraded. Together, RNA structures could affect their expression levels in cells, which always influences the discovery of potential novel ncRNAs.

### Principle and strategy for identification of novel ncRNAs

Nowadays, increased types of ncRNAs have been detected and identified by the development of next-generation sequencing (NGS) [58], which can be roughly divided into the process sections of sample preprocessing, library preparation, sequencing, and bioinformatics. Importantly, it shoud be noted that the ways of RNA isolation and library preparation greatly affect the detection of target species of ncRNAs.

Organic reagent method using isothiocyanate/phenol/chloroform or Trizol (Invitrogen) is an universial RNA extraction way to obtain total RNA containing small and intermediate-sized RNA. However, it has been reported that phenol contamination has influences on RNA yields and subsequent sequencing [110]. Spin column chromatography using commerial kits without phenol can avoid this contamination and obtain relatively high-quality RNA from the same samples. However, silica-based spin column chromatography fails to efficiently capture RNA shorter than 200 nt, which leads to massive loss of small and intermediate-sized ncRNAs and makes the way unsuitable for small RNA-seq [111, 112]. In contrast, the ways using spin column that can capture all RNA greater than 10 nt can be selected when we aim to obtain total ncRNAs or specifically enrich small ncRNAs. Choosing appropriate ways of RNA extraction is important for identification of novel ncRNAs with a specific size.

Library with appropriate RNA selection/depletion is also pivotal in the detection of specific types of ncRNAs. In library preparation for mRNA sequencing, RNAs with polyadenylated tails are specifically isolated by hybridization with poly(dT) oligomers from nonpolyadenylated RNAs which include a vast number of rRNAs. As a part of lncRNAs do not have polyadenylated tails, polyadenylated tail selection can only capture mRNA-like lncRNAs [113]. As for total lncRNA sequencing, library preparation is generally dependent on rRNA depletion methods. Next, the filtered RNAs are fragmented, reverse transcribed into cDNA by random primers, and undergo end repair, sequencing adaptor ligation, and size selection for subsequent sequencing. In this way, not only lncRNA but also mRNA, circRNA, and a part of intermediate-sized ncRNAs can be detected. However, reverse transcription (RT) by random primering and size selection leads to the deficiency of small ncRNAs such as miRNAs [114]. Depletion of linear RNAs by Rnase R treatment for circRNA sequencing and separation of RNAs with specific size by gel electrophoresis can specifically enrich target types of ncRNAs for RNA-seq, which are as far as possible to reduce interference signal from other transcripts. In addition, due to the shortened size, small RNA is hard to be successfully acquired through cDNA synthesis (first or second cDNA synthesis) by random priming and be always removed by size selection after sequencing adaptor ligation [114]. Thus, in small RNA-seq, both ends of the RNA fragments are firstly ligated to the adapters and followed by the cDNA synthesis and library construction. We also need to pay attention to the effects of RNA modifications on library preparation, which usually influence adapter ligation. For example, 5′ caps of snRNAs shoud be removed before adapter ligation. Selecting appropriate methods of library preparation is also important for identification of novel ncRNAs [101, 107].

It is worth noting that alternative splicing processes enable great complexity in transcripts from the same genomic regions [115]. For linear ncRNAs, various isoforms can be relatively easy to identify by RNA-seq. Nevertheless, despite the identification of circRNAs based on the junction site, extra sequence identification is still needed to determine the actual sequences of circRNAs because of potential circRNA variants being generated from a single gene locus [116]. This issue results from alternative splicing that occurs within circRNAs with multiple exons (Fig. 1c) [116]. All four basic types of canonical alternative splicing were found to occur in circRNAs as well: cassette exon, intron retention, alternative 5′ splicing and alternative 3′ splicing (Fig. 1c) [116]. For example, the human *XPO1* gene locus has been demonstrated to contain a circRNA-predominant cassette exon, the *CAMSAP1* gene locus generates two cirRNA isoforms with or without a retained intron, and the human *EIF3J* and *PAIP2* gene loci can also produce circRNAs containing both exon and intron sequences [104, 117, 118]. Other factors, such as read-through transcription and the fusion of genes derived from chromatin rearrangement, also generate read-through circRNAs and fusion circRNAs, respectively, which increase the diversity of ncRNAs [119, 120].

Traditionally well-known small noncoding RNAs, including miRNAs, siRNAs, and piRNAs, function in concert with the Argonaute (Ago) family of proteins to regulate gene expression at the level of transcription, mRNA stability, or translation [121, 122]. Interestingly, sdRNAs were initially discovered from an analysis of small RNAs associated with human Ago1 and Ago2 revealed by immunoprecipitation and RNA-seq (Fig. 1d) [10]. In addition, a number of novel small ncRNAs derived from both rRNAs (rRFs) and tRNAs (tsRNAs) have also been found to be enriched in RNA-induced silencing complexes (RISCs), and they function in a miRNA-like pathway (Fig. 1d) [9, 11, 36]. Immunoprecipitation of members of the Ago family proteins followed by small RNA-seq has revealed a series of novel small ncRNAs that play roles in RNA-induced target gene silencing. These data suggested that functional ncRNAs in well-known complexes should have more extensive sources and that transcripts derived from canonical DNA regions could have functions in addition to their classical ones by interacting with nonclassical RNA binding proteins (RBPs) or being located in novel complexes. This method of identifying RNA found in specific complexes or associating with subcellular components followed by RNA-seq represents an ideal way to discover new species of functional small ncRNAs. For example, the Vault complex, a novel ribonucleoprotein that probably functions in the nuclear export of large molecules, was isolated and characterized in 1986 [123]. By analyzing the components of Vaults, researchers discovered a novel and single species of small ncRNAs that is 86-141 nt in length, which was termed Vault RNAs (vRNAs) [124]. VRNAs that are derived from *VTRNA* genes by RNA polymerase III (Pol III) have been reported to be associated with multidrug resistance and, interestingly, also be the origin of miRNA-like small ncRNAs processed by Dicer [125]. Another example of identification or RNAs in complexes is snoRNAs, whose canonical functions are generally considered to guide the pseudouridylation and 2′-O-methylation of rRNA in the nucleolus [126]. However, in situ global RNA interactions with DNA identified by immunoprecipitation and RNA-seq showed that snoRNAs represent a vast population and a high enrichment in the chromatin-bound fractions, suggesting the other potential functions of these well-known small ncRNAs located in the nonclassical complexes [71, 127, 128].

Lack of sequence conservation, low level or high tissue-specific expression pattern, or derivation from canonical DNA sequences are potential factors that make the discovery and identification of novel ncRNAs difficult. We provided the identification principle of recently discovered functional ncRNAs, which would be a referential principle for novel ncRNA discovery. Importantly, recent technological developments, especially specific sequencing technological developments, have provided multiple approaches for the discovery and study novel ncRNAs.

### Approaches for discovering ncRNAs

Most ncRNAs, such as lncRNAs and circRNAs, have the characteristics of spatiotemporal specificity and low expression levels, which make it difficult to identify them [24, 102–104]. Therefore, it is necessary for us to purposefully choose the appropriate methods in sample preparation and sequencing techniques. Here, we will review innovative and novel sequencing methods that significantly improve the process of RNA identification and investigation, placing special emphasis on their advantages and limitations (Table 1).

### Tiling arrays and microarrays

Tiling array is an alternative and classic method for discovering RNA [56]. This approach hybridizes complementary DNAs (cDNAs) to microarray slides containing tiled oligonucleotide probes that are designed to hybridize with nonrepetitive sequences of specific genomic regions or the entire genome [56]. For example, tiling arrays were used to specifically identify the potential transcripts from four human *HOX* gene clusters with 400,000 probes, leading to the discovery of intergenic ncRNAs, including the well-known lncRNA HOX antisense intergenic RNA (HOTAIR) [129]. Tiling arrays can also provide in-depth analysis of alternative splicing, polymorphism, and novel transcription site identification by elevating the resolutions of designed probes [56, 130]. Nevertheless, because microarrays suffer from potential noise as a result of weak binding or cross-hybridization of transcripts to probes, tiling arrays have been replaced by NGS technologies and now preferably serve as a supplemental step for RNA-seq to increase the sequencing depth of target regions.

Microarray is an important method for performing quick global or parallel expression analysis of the transcriptome in different cell/tissue types, experimental systems, developmental stages, or pathological conditions [57]. This classic method consists of a large number of oligonucleotide probes spotted on a solid surface that are then allowed to hybridize to target sequences from samples, which are further detected by fluorescently labeled target sequences. The intensity of fluorescence is used to quantify target sequences. Their small size and high-throughput capabilities have brought microarrays to the forefront of RNomic research. However, this approach can only detect RNAs whose sequences are known and have specific hybridization probes; this method is not able to discover novel transcripts.

### RNA-seq

**Fig. 2:**
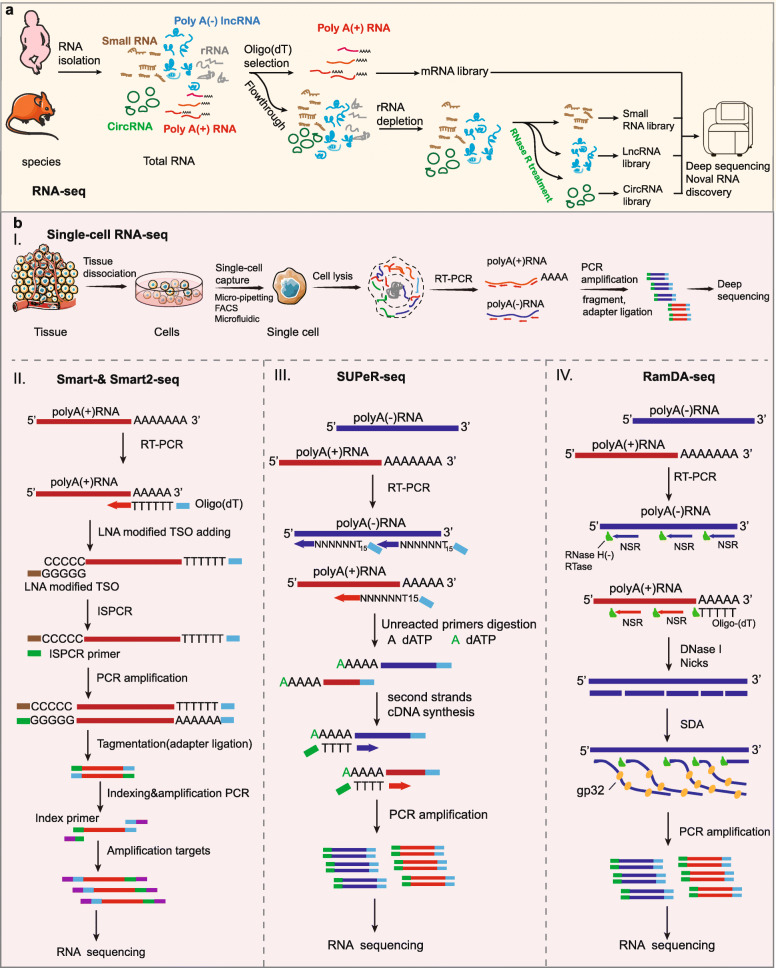
Technologies for novel ncRNA discovery. **a** Process diagrams of RNA-seq. RNA-seq with purposeful experimental treatments can be used to detect diverse species of ncRNAs, including lncRNAs, circRNAs, and small ncRNAs. **b** Process diagrams of scRNA-seq. (I) The schematic of single-cell RNA-seq. Single cells are isolated and lysed to release total RNAs. RNAs are then reverse transcribed into first-strand cDNAs using designed primers followed by amplification for RNA-seq. (II–IV) The detailed schematic of innovative and novel methods such as Smart-seq (II), SUPeR-seq (III), and RamDA-seq (IV). In Smart-seq, polyadenylated RNAs are reverse transcribed into a pool of cDNAs by oligo (dT) primers followed by adding nontemplate C nucleotide tails to the 3′ ends (II); however, SUPeR-seq uses random primers with fixed anchor sequences for cDNA synthesis, followed by adding poly(A) tails to the 3′ ends (III). (IV) RamDA-seq uses both oligo (dT) and random primers for cDNA synthesis. cDNA is synthesized by the RNA-dependent DNA polymerase activity of RNase H minus reverse transcriptase (RTase). DNase I selectively nicks the cDNA of the RNA:cDNA hybrid strand. The 3′ cDNA strand is displaced by the strand displacement activity of RTase mediated by the T4 gene 32 protein (gp32), starting from the nick randomly introduced by DNase I. cDNA is amplified and protected by gp32 from DNase I. NSR: not-so-random primer

RNA-seq is currently the most widespread sequencing technology for both detecting RNA expression and discovering novel species of ncRNAs (Fig. 2a) [24, 58]. In addition, this approach can also be used to identify single nucleotide polymorphisms, alternative splicing isoforms, gene fusion events, and novel splice junctions [131–134]. RNA-seq is based on the conversion of RNA into a pool of cDNA with either oligo (dT) primers or random primers, depending on the purpose of the sequencing. However, because cDNA libraries prepared with oligo (dT) selectively enrich for polyadenylated RNA and simultaneously deplete nonpolyadenylated and partially degraded transcripts, RNA-seq with random primers for cDNA synthesis on rRNA-depleted transcripts is currently a more widely used approach. Analysis of human or mouse cell types using RNA-seq revealed the presence of more than 8000 human and over 1000 mouse long intergenic ncRNAs (lincRNAs), the majority of which had not been previously identified [32, 135]. Interestingly, in one study using RNA-seq for the specific identification of nonpolyadenylated RNA, a novel species of lncRNAs with snoRNA-like ends was discovered to be produced from excised introns [42, 105]. Moreover, the first identification of large numbers of circRNAs in humans and mice occurred following the combination of RNA-seq and RNase R treatment, which uncovered the effective presence of 1950 human and 1903 mouse circRNAs in human cell lines (HEK293 and leukocytes) and mouse tissues such as the brains and fetal heads [7]. In addition, RNA-seq with specific preparation for small RNA identification is also the primary approach for discovering and detecting miRNAs, snoRNAs, piRNAs, and other novel small ncRNAs, including IRFs, tsRNA, and sdRNAs [10, 126, 136–139].

There is a derivative technology based on RNA-seq, RNA capture sequencing, which is combined with tiling arrays to elevate the sequencing depth of target regions [59]. In brief, tiling arrays are performed first with specific oligonucleotide probes to enrich cDNAs from specific genomic regions. Second, the hybridized cDNAs are eluted and then sequenced by RNA-seq. RNA capture sequencing increases the sequencing depth in specific genomic regions compared to RNA-seq and has uncovered multiple unannotated isoforms of both mRNAs and ncRNAs, including a novel alternative splicing transcript of HOTAIR that lacks the binding domain for the polycomb repressive complex (PRC2) [59].

Over the years, many technologies based on basic RNA-seq have been developed to identify RNAs at the transcriptome scale, some of which will be discussed in the following sections. It is inferred that advanced algorithms for analysis of sequencing data are also likely to promote transcriptome analysis. Nevertheless, RNA-seq may suffer from disadvantages such as the introduction of sequence errors at the reverse-transcription step or primer bias, which require further optimization [140].

### Small RNA-seq and single-cell small-RNA sequencing

Because sample preparation for RNA-seq is not suitable for small RNAs, such as reverse transcription with random priming (short RNA species yield even shorter cDNAs that are not long enough for efficient alignment), small RNA-seq with modified library preparation, such as miRNA-seq, was developed [65, 114, 141]. Small RNA-seq is a type of RNA-seq that discriminate small RNAs from larger RNAs to better evaluate and discover novel small RNAs [65]. In this method, small RNAs are fractionated by gel electrophoresis, and then, universal adapters are ligated to the both ends of RNA fragments, which are acted as primer binding sites during reverse transcription and PCR amplification. Previous studies using small RNA-seq detect specific expression profiles of miRNAs in various biological processes and cancer; reveal asymmetric processing of small RNAs from rRNAs, snoRNAs, snRNA, and tRNAs; and even provide evidence for human miRNA-offset RNAs [65, 142, 143]. Although adapter ligation bias which lead to reverse transcription bias or amplification bias still need to be optimized [144, 145], small RNA-seq currently remains a high-efficiency way to detect and discover novel small ncRNAs.

A recent study provided a method to detect small ncRNAs in a single cell and the method was named as Small-seq [66]. In brief, single cell is lysed, and 5.8S rRNA is masked with a complementary oligonucleotide during adapter ligation. Then 3′ adapters are ligated to small RNAs, and unligated adapters are subsequently digested. The 5′ adapters containing a unique molecular identifier (UMI) are ligated, and reverse transcription is carried out. In the original article, the method captured a complex set of small RNAs, including miRNAs, fragments of tRNAs, and snoRNAs [66].

### Single-cell RNA sequencing (scRNA-seq)

The fundamental unit of an organism is a single cell. Along with in-depth studies on development and disease occurrence, there is a growing sense that some single cells possess nonnegligible abilities that can affect organic growth or lead to the downfall of the entire organism [146]. It is helpful for researchers to further understand the mechanisms of growth or disease progression by revealing the gene expression pattern of specific single cells. However, the sample sizes from a single cell are insufficient for general RNA-seq, which has led to the development of scRNA-seq methods (Fig. 2b(I)). In addition, scRNA-seq techniques are also appropriate for other small samples, such as limited clinical patient samples or cells sorted with fluorescence-activated cell sorting (FACS) [61, 147].

Previous scRNA-seq techniques include Smart-seq [60, 148], designed primer-based sequencing (DP-seq) [61], and Quartz-seq [62], and each of them exhibits prominent advantages and disadvantages. Smart-seq is a method based on a full-length cDNA amplification strategy (Fig. 2b(II)) [60]. In this approach, polyadenylated RNAs are reverse transcribed into a pool of cDNAs by oligo (dT) primers and Moloney murine leukemia virus reverse transcriptase (MMLV RT). As a result, the terminal transferase activity of MMLV can add several nontemplate C nucleotides to the 3′ ends of the reverse transcribed products when the reverse transcription reaction reaches the 5′ end of a template transcript during first-strand cDNA generation (Fig. 2b(II)). Then, the poly-cytidine overhangs are used to complete the double-strand cDNA generation, which ensures that the prepared library for scRNA-seq only contains full-length cDNAs. However, the lack of strand-specific identification and inability to read transcripts longer than 4 kb partly limit the application of this method [149]. Compared to Smart-seq, DP-seq shows the advantage of being to amplify RNAs from smaller size samples, as low as 50 pg, or from transcripts longer than 4 kb [61]. DP-seq uses a defined set of heptamer primers, which target regions less likely to form secondary structures and reside upstream of the unique regions on certain transcriptomes, and they amplify the majority of expressed transcripts from a limited number of RNAs [61]. In the original study, preparation of a DP-seq library successfully amplified over 80% of the mouse transcriptome with 44 heptamer primers. Moreover, this approach can also suppresse highly expressed rRNAs in the cDNA library and is able to detect transcripts at relatively low levels [61]. In addition, Quartz-seq is an alternative scRNA-seq method with reduced background noise that utilizes specially designed suppression polymerase chain reaction (PCR) primers to reduce the generation of unwanted side products [62].

Recent studies on scRNA-seq methods preferably focused on total RNA sequencing, which provided rich information on biological systems in addition to the abundance of mRNAs. Thus far, much efforts have been made to develop scRNA-seq techniques with full-length coverage or sensitivity to nonpolyadenylated RNAs. There are several scRNA-seq methods, such as Smart-seq, that can provide full-length coverage of transcripts [60]. Nevertheless, these methods fail to measure nonpolyadenylated transcripts due to oligo (dT) priming [60]. Single-cell universal poly(A)-independent sequencing (SUPeR-seq), which uses random primers with fixed anchor sequences to replace oligo (dT) primers for cDNA synthesis, has been reported for the detection of nonpolyadenylated RNAs, especially circRNAs, in a single cell with robust precision and accuracy (Fig. 2b(III)) [63]. In the original study, researchers discovered 2891 circRNAs and 913 novel linear RNAs in mouse preimplantation embryos using SUPeR-seq and deciphered regulation mechanism of circRNA during early embryonic development [63]. However, SUPeR-seq also exhibits relatively low sensitivity for nonpolyadenylated RNAs [64].

Random displacement amplification sequencing (RamDA-seq) is a full-length total RNA-sequencing method for analyzing single cells, but it has a high sensitivity for nonpolyadenylated RNAs [64]. This approach can measure not only polyadenylated but also nonpolyadenylated RNAs, including nascent RNAs, lncRNAs, circRNAs, and eRNAs, and it can uncover the dynamics of recursive splicing [64]. Furthermore, it can provide full-length coverage for extremely long transcripts (more than 10 kb). RamDA-seq simplifies the experimental procedure to amplify cDNA as early as possible by using a novel RT technology, RT with random displacement amplification (RT-RamDA), which aims to obtain higher capture efficiency of RNAs and global cDNA amplification for further sequencing (Fig. 2b(IV)). Moreover, not-so-random primers (NSRs) are used to enable random priming while preventing the synthesis of cDNA from rRNAs [64]. Analysis of mouse embryonic stem cells undergoing differentiation using RamDA-seq revealed the cell state-dependent expression of known and novel nonpolyadenylated RNAs, including nonpolyadenylated isoforms of the lncRNA Neat1, and the specific genome-wide eRNA expression patterns in single cells [64].

### Nascent RNA-seq

RNA-seq is a revolutionary tool for transcriptome profiling that provides information on the dynamic changes of gene expression against different conditions or after exposure to different stimuli [58]. However, the traditional RNA-seq technique is generally performed to determine steady-state RNA levels, and changes in RNA transcription and decay rates cannot be easily distinguished [150]. Moreover, common RNA-seq also fails to provide efficient temporal information on RNA kinetics [150]. To address these issues, new sequencing methods for measuring nascent transcripts, as opposed to total RNAs, have been developed [151].

**Fig. 3:**
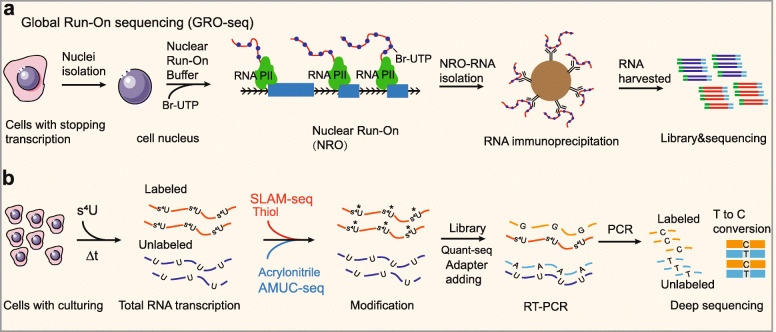
Process diagrams of representative nascent RNA-seq methods. **a** Schematic of GRO-seq. In this approach, nascent RNAs are labeled with 5Br-UTP and immunoprecipitated with the antibody anti-Br-UTP; the isolated RNAs subsequently undergoes deep sequencing. **b** Schematic of methods based on base mutation for nascent RNA detection. Nascent RNAs are labeled with a thiol-labeled nucleoside (s^4^U or s^6^G), and these newly synthesized RNAs can then be isolated and treated with specific chemical reagents, such as thiol (SLAM-seq) and acrylonitrile (AMUC-seq), leading to a change in the base-pairing manner of metabolically incorporated nucleosides

Nascent RNA-seq can reveal the temporal information of gene expression changes. Metabolic labeling and affinity purification of labeled nascent RNAs followed by RNA-seq is a well-known approach for analyzing nascent RNAs [151]. For example, global run-on sequencing (GRO-seq) labels nascent RNAs with 5Br-UTP, enabling labeled nascent RNAs to be immunoprecipitated with the antibody anti-Br-UTP; the isolated RNAs subsequently undergoes deep sequencing (Fig. 3a) [67]. By sequencing nascent RNAs, GRO-seq can also provide a genome-wide view of the location, orientation, and density of Pol II-engaged transcripts, revealing divergent transcription at active promoters that yield antisense ncRNAs [152]. In recent studies, labeling/purifying RNA analysis has also been used to detect nascent ncRNAs, including nascent circRNAs. Nevertheless, the conventional purification assay in GRO-seq is confounded by contamination due to nonspecific binding, which could possibly result in experimental bias [70].

Recently, innovative enrichment-free methods for nascent RNA detection have been developed, which avoid contamination induced by affinity purification [153]. These methods directly distinguish nascent RNA from total RNA in single-base resolution by marking the mapping reads of nascent RNAs with introduced base mutations. In brief, nascent transcripts are labeled by adding a thiol-labeled nucleoside (s^4^U or s^6^G) to cell culture media, and these newly synthesized RNAs can then be isolated and treated with specific chemical reagents, leading to a change in the base-pairing manner of metabolically incorporated nucleosides (Fig. 3b) [153]. For example, SLAM-seq uses nucleophilic substitution chemistry to induce s^4^U-to-C conversion in an RT-dependent manner [68], and TimeLapse-seq employs s^4^U-to-C conversion via an oxidative nucleophilic aromatic substitution reaction (Fig. 3b) [69]; however, this oxidation condition caused certain oxidative damage to guanine [69]. A recent study reported an improved method, AMUC-seq, which transformed s^4^U into a cytidine derivative using acrylonitrile (Fig. 3b) [70]. Compared to other enrichment-free methods for nascent RNA detection, AMUC-seq has been reported to be more efficient and reliable because it has a minimal influence on the base-pairing manner of other nucleosides and can quantitatively analyze RNA at the transcriptome scale [70].

### Innovative techniques based on RNA location and interactome for functional ncRNA discovery

As discussed above, the vast majority of the human genome can be transcribed into ncRNAs; thus, it is important to reveal potentially functional ncRNAs that may play a role in certain biological processes, especially in cancer occurrence and development. It has been shown that ncRNAs are commonly folded into highly ordered structures that play a role within their interactome [154, 155]. Therefore, in this section, we will discuss the discovery and identification of functional ncRNAs based on their interaction networks and subcellular location levels, and we will provide some novel techniques that can be used to screen purposefully for functional ncRNAs.

### RNA-chromatin interaction

**Fig. 4:**
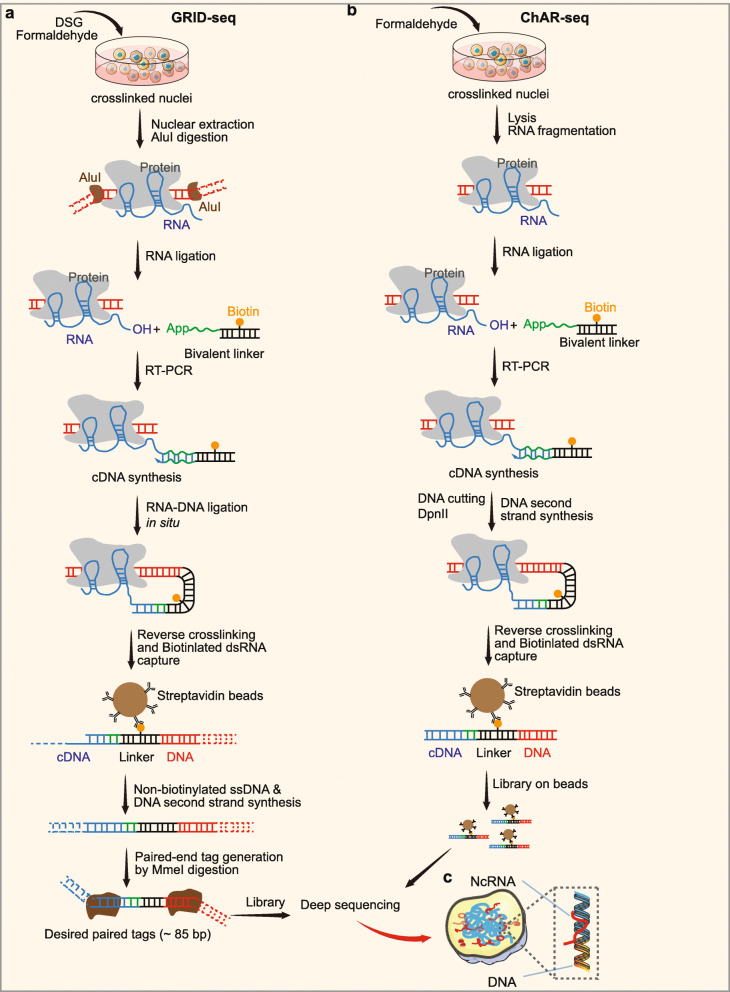
Technologies for discovery of RNA-chromatin interaction. **a** Process diagrams of GRID-seq. Cells are fixed with disuccinimidyl glutarate (DSG) and formaldehyde. Then, nuclei are extracted, and DNA is digested in situ by the frequent 4-base cutter AluI. A specifically designed bivalent linker labeled by biotin that consists of single-stranded RNA (ssRNA) portions, to ligate RNA, and a double-stranded DNA (dsDNA) portion, to ligate DNA, is used to link RNAs to AluI-digested genomic DNAs. DNA ligation to AluI-digested genomic DNA are performed in situ followed by affinity purification on streptavidin beads. Then, ssDNA are released from the beads, generated into dsDNA, cleaved by a type II restriction enzyme MmeI and sequenced. **b** Overview of the ChAR-seq method. RNA-DNA contacts are preserved by crosslinking, followed by in situ ligation of the 3′ end of RNAs to the 5′ end of the ssDNA tail of a bivalent linker containing biotin and a DpnII-complementary overhang on the opposite end. After generating a strand of cDNA complementary to the RNA, the genomic DNA is then digested with DpnII and then re-ligated, capturing proximally associated bridge molecules and RNA. The chimeric molecules are reverse transcribed, purified, and sequenced

An increasing number of studies have reported that diverse species of ncRNAs show regulatory functions in different layers of and gene expression. Many cnRNAs perform direct actions on chromatin, some of which may mediate genomic interactions predominantly in *cis*, whereas others are capable of acting extensively in *trans* [156–158]. These findings suggest a common role of specific RNA-chromatin interactions in modulating gene expression. Global RNA interactions with DNA by deep sequencing (GRID-seq) is a method that aims to comprehensively detect and determine the localization of all potential chromatin-interacting RNAs [71]. This approach uses a bivalent linker to ligate RNA to DNA in situ in fixed nuclei (Fig. 4a). Briefly, cells are fixed with disuccinimidyl glutarate (DSG) and formaldehyde first to stabilize RNAs on chromatin. Then, nuclei are extracted, and DNA is digested in situ by the frequent 4-base cutter AluI. A specifically designed bivalent linker labeled by biotin that consists of single-stranded RNA (ssRNA) portions, to ligate RNA, and a double-stranded DNA (dsDNA) portion, to ligate DNA, is used to link RNAs to AluI-digested genomic DNAs. Then, the DNA-RNA complexes are purified, filtered, and sequenced. In the original article, GRID-seq performed in human, mouse, and *Drosophila* cells revealed a large set of tissue-specific coding and noncoding RNAs that bind to active promoters and enhancers, especially super-enhancers [71]. Interestingly, the study also exhibited a large number of snoRNAs interacting with chromatin, suggesting possibly important roles of snoRNAs at the chromatin level [71].

Other alternative techniques based on the ligation of RNA to DNA have been reported for detecting genome-wide RNA-chromatin interactions, including MARGI and its improved version iMARGI [72, 159], and chromatin-associated RNA sequencing (ChAR-seq) [73]. Analysis of chromatin-associated RNA (caRNA) sequencing by MARGI and iMARGI revealed that caRNAs not only are associated with genomic regions where they are generated (proximal interactions) but also are attached to distal genomic regions (distal interactions) on the same chromosomes or on other chromosomes (interchromosomal interactions) [72, 159]. Interestingly, transcription star sites (TSSs) were identified as the preferred genomic regions targeted by chromatin-associated ncRNAs through distal or interchromosomal interactions. ChAR-seq also uncovered a range of chromatin-associated RNAs, especially chromosome-specific dosage compensation ncRNAs, and genome-wide trans-associated RNAs, which are involved in cotranscriptional RNA processing (Fig. 4b) [73].

In addition to the sequencing methods for identification of global RNA-chromatin interactomes mentioned above, various techniques were developed to detect specific localization on chromatin of target RNAs [160–162]. These techniques use hybridization of complementary oligonucleotides to pull down a single target RNA, and then NGS or mass spectrometry is performed to identify its DNA- or protein-binding partners.

### RNA-RNA spatial interactions

**Fig. 5:**
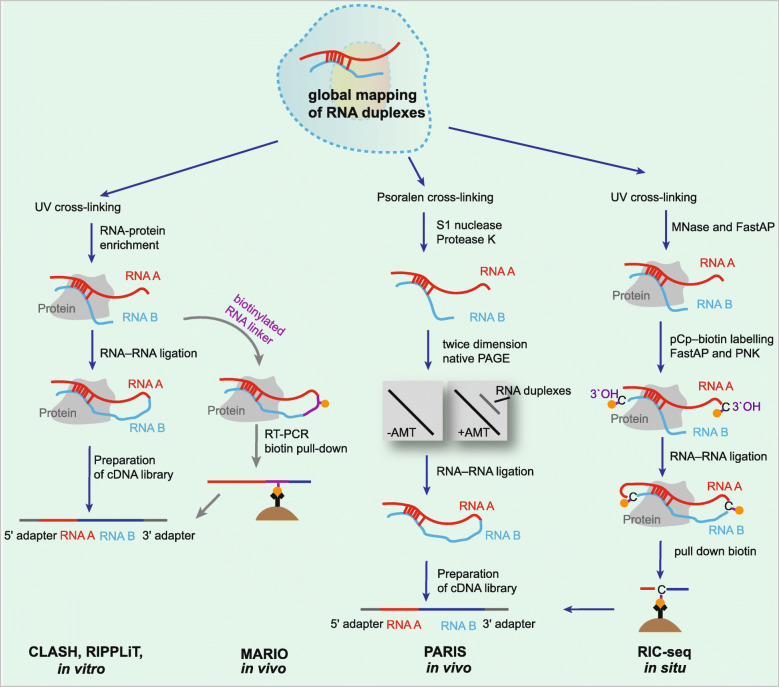
Technologies for capturing RNA secondary structures and tertiary interactions. Schematics of CLASH, RIPPLiT, MARIO, RARIS, and RIC-seq. MNase, micrococcal nuclease

Structured RNAs such as duplexes represent a feature that is critical for most steps in the gene expression pathway. Numerous characterized ncRNAs function via base pairing with target RNAs to control their biological activities, such as dynamic interactions involving snRNA-snRNA and snRNA-pre-mRNA during the assembly and disassembly of spliceosomes, interactions between snoRNAs and their target RNAs to guide RNA modification, and interactions between ncRNAs and mRNAs that regulate transcript turnover and translation. Thus far, an increasing number of sequencing techniques have been developed for global mapping of RNA-RNA interactions (Fig. 5).

RNA proximity ligation is a set of molecular biological techniques that can be used to analyze the conformation and spatial proximity of RNAs in cells [74]. The typical first steps in these approaches involves cross-linking biological samples with UV light or psoralen, which is followed by partial fragmentation of RNA, RNA-RNA ligation, library preparation, and high-throughput sequencing. UV light and psoralen are two widely used methods for sample preparation prior to proximity ligation: UV light treatment stabilizes and enriches the RNA duplexes that are bound to a specific protein or protein complex; however, psoralen is used to stabilize and enrich RNA-RNA interactions. Studies on RNA conformation have shown different emphases, as some approaches identified pairs of RNAs that are in direct contact or in close proximity with each other, while others recovered pairs of RNAs that are part of the same protein complex or subcellular compartment [163]. Alternative cross-linking methods provide alternative treatments for diverse purposes (Fig. 5). Cross-linking ligation and sequencing of hybrids (CLASH) is a relatively early method that uses UV cross-linking to capture direct RNA-RNA hybridization [74]. Compared to chemically cross-linking methods, which also induce extra protein-protein cross-linking, CLASH has the advantage of avoiding noise from protein intermediate-mediated interactions, and has been used to identify novel snoRNA-rRNA interactions in yeast [74], miRNA-mRNA interactions in human HEK293 cells [164], and piRNAs interactomes [164]. In another method, RNA immunoprecipitation and proximity ligation in tandem (RIPPLiT), sequential pull-down of components of exon junction complexes showed a mapping of mRNA conformations when bound to this complex [75]. Moreover, another approach, mapping the RNA interactome in vivo (MARIO), has identified RNA-RNA interactions in the vicinity of all RNA-binding proteins using a biotin-linked reagent [76].

Methods for identifying RNA-RNA interactions at the transcriptome scale by cross-linking with psoralen have been reported since 2016 [77, 79, 165]. Unlike CLASH, psoralen-based approaches do not depend on the pull-down of RNA-RNA interactions with a specific protein, and in principle, they can yield transcriptome-wide RNA interactomes. The methods using this principle of cross-linking RNAs are combined with different means to enrich cross-linked fragments, such as two-dimensional gel electrophoresis in PARIS (psoralen analysis of RNA interactions and structures) [77], digestion by RNase R in LIGR-seq (ligation of interacting RNA followed by high-throughput sequencing) [78], and biotin-streptavidin enrichment in SPLASH (sequencing of psoralen cross-linked, ligated, and selected hybrids) [79]. The psoralen cross-linking methods uncovered general properties of RNA-RNA interactomes in mammalian cells (Fig. 5). For example, PARIS uncovered alternative base pairing in intramolecular interactions, which suggests substantial structural heterogeneity in cells, and it also elucidated the structure produced by a repeat of adenosines in Xist in vivo [77]. LIGR-seq in HEK293 cells detected novel snRNA-snRNA and snoRNA-rRNA interactions [78]. More importantly, this approach also revealed that SNORD83B can regulate gene expression by binding to target mRNAs, revealing an unexpected function of these snoRNAs [78]. Psoralen cross-linking methods such as PARIS and SPLASH were also applied to detect dense networks of RNA-RNA interactions within viral genomes inside infected cells [166, 167].

A recent study reported a novel method, RNA in situ conformation sequencing (RIC) technology, for the global mapping of intra- and intermolecular RNA-RNA interactions (Fig. 5) [80]. Compared to the RNA ligation induced in vitro in previous methods, RIC-seq performs RNA proximity ligation in situ, and it enriches chimeric reads using a biotinylated cytidine phosphate (pCp-biotin) [80]. Briefly, the cells are cross-linked by formaldehyde, and then, RNA is randomly cut with micrococcal nuclease and dephosphorylated at 3′ overhangs. The 3′ ends are labeled with pCp-biotin and ligated to proximal 5′ overhangs under in situ and nondenaturing conditions. Total RNAs are fragmented in vitro, and RNAs containing C-biotin are enriched followed by conversion into cDNA libraries for sequencing. In the original article, RIC-seq was used to facilitate the generation of 3D RNA interaction maps in human cells, and it revealed global noncoding RNA targets, RNA topological domains, and trans-interacting hubs [80].

In addition to the sequencing methods using RNA proximity ligation, there are some other approaches without ligation that have been developed because of the possible limitation in efficiency of enzymatic ligations affected by short-range distances between RNA ends [81]. RNA proximity sequencing is a method based on massive-throughput RNA barcoding of particles in water-in–oil emulsion droplets [81]. In brief, this approach uniquely barcodes RNA in millions of subnuclear particles in parallel by a rapid vortexing step that combines fragmented nuclear particles with barcoded beads in a water-in-oil emulsion; then, the cDNA is sequenced. The detection of multiple RNAs in proximity to each other by RNA proximity sequencing distinguished RNA-dense and RNA-sparse compartments, and this technique is an alternative approach for studying the spatial organization of transcripts in the nucleus, including ncRNAs and their functional relevance.

### RNAs in protein complexes or subcellular structures

The location of ncRNAs in cells is the primary determinant of their molecular functions. NcRNAs, especially lncRNAs, are often considered as chromatin-restricted modulators of gene transcription and chromatin structure [157, 158]. In addition, a rich population of cytoplasmic ncRNAs, such as extra lncRNAs and exonic circRNAs, have been reported to play roles in diverse biological processes, including translational regulation and signal transduction [8, 168]. Elution-based methods promise to detect RNAs at the transcriptome scale associated with all organelles of mammalian cells, and RNA maps of increasing resolution reveal a subcellular world of highly specific localization patterns.

**Fig. 6:**
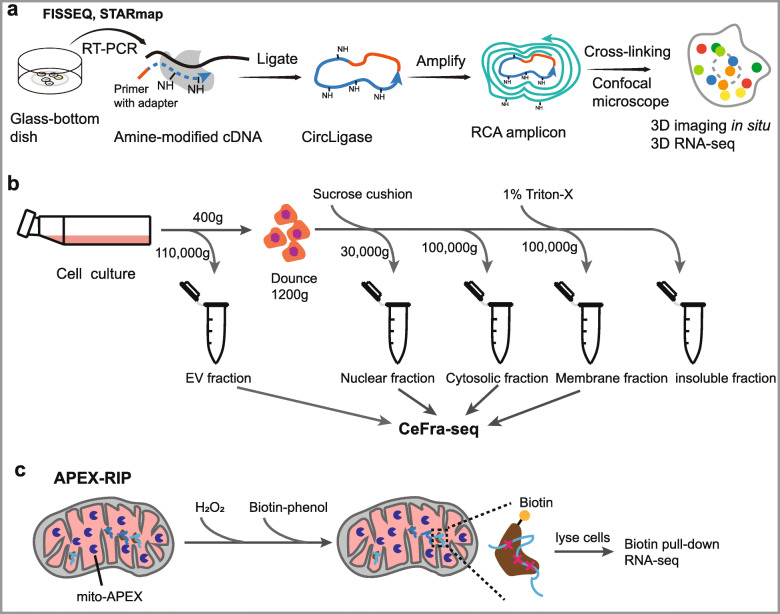
Technologies for discover of RNA location. **a** Schematics of FISSEQ and STARmap. FISSEQ begins with fixing cells on a glass slide and performing reverse transcription in situ with aminoallyl-dUTP and adapter sequence-tagged random hexamers. After RT, cDNA fragments are circularized at 60 °C. The circular templates are amplified using rolling-circle amplification (RCA) primers complementary to the adapter sequence in the presence of aminoallyl-dUTP and stably cross-linked. The nucleic acid amplicons in cells are then ready for sequencing and imaging. STARmap begins with labeling of cellular RNAs by pairs of DNA probes followed by enzymatic amplification so as to produce a DNA nanoball (amplicon). Tissue can then be transformed into a 3D hydrogel DNA chip by anchoring DNA amplicons via an in situ—synthesized polymer network. **b** Schematics of biochemical cell fractionation. Biological extracts including intact organelles are separated by density gradient or immunoprecipitation with specific antibodies. **c** Overview of the APEX-RIP. Cells expressing APEX2 targeted to the mitochondrial are cultured with the APEX substrate biotin-phenol. H_2_O_2_ initiates biotinylation of proximal endogenous proteins, which are subsequently crosslinked to nearby RNAs by formaldehyde. After cell lysis, biotinylated species are enriched by streptavidin pull-down, and coeluting RNAs are analyzed by RNA-Seq

In situ hybridization (ISH) is the most widely used method of RNA localization using labeled complementary oligonucleotide probes to visualize target RNAs [169, 170]. Single-molecule fluorescence ISH (smFISH) uses multiple probes to amplify the fluorescent signal for the detection of target RNAs at low levels, and it is thought of as the gold-standard technique for single-gene studies [169, 171]. In contrast to RNA smFISH, fluorescent in situ RNA sequencing (FISSEQ) offers in situ information at high-throughput levels [82]. In this approach, RNA is reverse transcribed in situ into cDNA in cross-linked cells and tissue samples, which is then analyzed by sequencing (Fig. 6a). However, compared to standard RNA-seq, FISSEQ also comes at the expense of lower read coverage, which reduces sensitivity for lowly expressed RNAs, especially ncRNAs [82]. Another alternative related technique, spatially resolved transcript amplicon readout mapping (STARmap), provided 3D locational information of RNA expression in intact tissue samples [172].

Biochemical cell fractionation is a fractionation-based method that physically isolates subcellular compartments and identifies their RNAs (Fig. 6b). These types of methods can be based on protein immunoprecipitation, intact organelle purification, or partitioning through sucrose gradients [173]. Then, RNA-seq (biochemical cell fractionation combined with RNA-seq, CeFra-seq) was performed to detect specific RNAs at the transcriptome scale [83]. Fractionation-based methods have high sensitivity for low-abundance transcripts due to aggregation across many cells; however, they are restricted by isolation protocols and the purity of resulting isolates, which possibly induce technical noise by contamination across fractions [24, 174, 175].

Recently, innovative techniques have been developed to overcome the deficiencies of conventional methods. A new fractionation-based method, APEX-RIP [84], was developed, and it combines APEX (engineered ascorbate peroxidase)-catalyzed proximity biotinylation [176] and RNA immunoprecipitation (RIP) [177] to map RNAs at vastly improved spatial resolution (Fig. 6c). In brief, APEX-catalyzed proximity biotinylation is targeted by genetic fusion to proteins from various subcellular compartments of interest. This is followed by protein-RNA crosslinking and RIP to pull down the biotinylated subcellular fraction for further high-throughput sequencing. Using this method, thousands of ncRNAs have been mapped to specific compartments without the need for purification of specific organelles, and it offers high specificity and sensitivity in targeting the transcriptome of membrane-bound organelles [84]. Moreover, in a recent study, a transcriptome-wide subcellular RNA atlas was generated by APEX-RIP [178].

### NcRNA database

Various sequencing methods have provided systematic expression profiling of ncRNAs in diverse cells, tissues, and organisms, and they have mapped the interaction networks or subcellular localization of ncRNAs, which inform their potential biological functions. Databases provide important references based on theoretical analysis, sequencing data, and even experimental verification, which play a guiding role in the identification and functional investigation of ncRNAs. Here, we will introduce a series of ncRNA databases that emphasize basic ncRNA information, cancer-associated ncRNA expression patterns, or specific ncRNA interaction networks based on experimental techniques followed by high-throughput sequencing.

The correlations between ncRNA expression and cancer progression provide important hints whether a ncRNA could play a role in certain cancers. There are an increasing number of databases providing comprehensive associations between ncRNAs and human cancers, which are supported by sequencing data or even experiments, such as TANRIC [179], Lnc2Cancer 2.0 [180], lnCaNet [181], and LncRNADisease [182] for lncRNAs, CSCD [183], Circ2Traits [184], CircR2Disease [185], and MiOncoCirc [119] for circRNAs, miRCancer [186], SomamiR 2.0 [187], OncomiR [188], miRCancerdb [189], and dbDEMC 2.0 [190] for miRNAs and YM500v3 [191], tRF2Cancer [192], and MINbase v2.0 [193] for other small ncRNAs, as summarized in Table 2. A recently reported MiOncoCirc is the first database that mainly consists of circRNAs directly detected in tumor tissues [119]. It was established by detecting and characterizing circRNAs across more than 2000 cancer samples with an exome capture RNA sequencing protocol. In the article that originally described the process, candidate circRNAs identified from MiOncoCirc were determined to be useful as biomarkers for prostate cancer and were found to be detected in urine, suggesting that MiOncoCirc could be an alternative tool to uncover novel diagnostic biomarkers for clinical translational strategies [119]. Another interesting, recently reported database is SELER, which collects specific super-enhancer-associated lncRNA profiles from different cancers [195]. In addition, some databases document the basic annotation and functional information on ncRNAs, including lncRNA-associated resources LNCipedia [199], LNCediting [200], lncRNAdb v2. 0[201], circRNA-associated ones circAtlas [206], circBase [207], CIRCpedia v 2[208], TSCD [209], miRNA-associated ones starBase v2.0 [210], miRTarBase [211], miRmine [212], EVmiRNA [213], miRGate [214], miRBase [215], and even other small ncRNA-associated ones DASHR 2.0 [217]. A growing number of databases have undoubtedly played important roles in the discovery and investigation of novel functional ncRNAs.

**Table 2:** Database of ncRNAs

Cancer or basis	Database	Species	Website	Short description	Ref
Cancer	Lnc2Cancer v2.0	lncRNA	http://www.bio-bigdata.net/lnc2cancer	An updated database that provides comprehensive experimentally supported associations between lncRNAs and human cancers.	[180]
	TANRIC	lncRNA	http://bioinformatics.mdanderson.org/main/TANRIC:Overview	This database characterizes the expression profiles of lncRNAs in large patient cohorts of 20 cancer types, including TCGA and independent datasets (> 8000 samples overall).	[179]
	lnCaNet	lncRNA	http://lncanet.bioinfo-minzhao.org/	This database provides a comprehensive co-expression data resource which reveals the interactions between lncRNA and non-neighbouring cancer genes.	[181]
	LncRNADisease 2.0	lncRNA	http://www.rnanut.net/lncrnadisease/	A database integrating comprehensive experimentally supported and predicted lncRNA-disease associations.	[182]
	The Cancer LncRNome Atlas	lncRNA	http://tcla.fcgportal.org/	An academic research database to explore the lncRNA alternations across multiple human cancer types.	[194]
	SELER	lncRNA	http://www.seler.cn/download.php	A database of super-enhancer-associated lncRNA-directed transcriptional regulation in human cancers.	[195]
	CSCD	circRNA	http://gb.whu.edu.cn/CSCD	A database that focuses on distinguishing cancer-specific circRNAs from noncancerous circRNAs, and reports predicted cellular location, RBP sites, and ORFs.	[183]
	Circ2Traits	circRNA	http://gyanxet-beta.com/circdb/	Provide cirRNA-disease association based on the interaction of circRNAs with disease-related miRNAs and SNP mapped on circRNA loci.	[184]
	CircR2Disease	circRNA	http://bioinfo.snnu.edu.cn/CircR2Disease/	Provide a comprehensive resource for circRNA deregulation in various diseases, containing 725 associations between 661 circRNAs and 100 diseases.	[185]
	CircRNA disease	circRNA	http://cgga.org.cn:9091/circRNADisease/	A manually curated database of experimentally supported circRNA-disease associations.	[196]
	MiOncoCirc	circRNA	https://nguyenjoshvo.github.io/	circRNA detection in 2093 clinical human cancer samples using exome capture sequencing.	[119]
	CircRiC	circRNA	https://hanlab.uth.edu/cRic	A database focusing on lineage-specific circRNAs in 935 cancer cell lines including drug response.	[197]
	miRCancer	miRNA	http://mircancer.ecu.edu/	A database currently documents more than 9000 relationships between 57,984 miRNAs and 196 human cancers.	[186]
	SomamiR 2.0	miRNA	http://compbio.uthsc.edu/SomamiR/	A database of cancer somatic mutations in microRNAs (miRNA) and their target sites that potentially alter the interactions between miRNAs and competing endogenous RNAs (ceRNA).	[187]
	OncomiR	miRNA	http://www.oncomir.org/	An online resource for exploring miRNA dysregulation in cancer.	[188]
	miRCancerdb	miRNA	https://mahshaaban.shinyapps.io/miRCancerdb/	An easy-to-use database to investigate the microRNAs-dependent regulation of target genes involved in development of cancer.	[189]
	miR2Disease	miRNA	http://www.miR2Disease.org	A database aiming at providing a comprehensive resource of microRNA deregulation in various human diseases.	[198]
	YM500v3	small ncRNA	http://ngs.ym.edu.tw/ym500/	A database which contains more than 8000 small RNA-seq dataseta and focuses on piRNAs, tRFs, snRNAs, snoRNAs, and miRNAs.	[191]
	tRF2Cancer	small ncRNA	http://rna.sysu.edu.cn/tRFfinder/	A web server to detect tRFs and their expression in multiple cancers.	[192]
	MINTbase v2.0	Small ncRNA	https://cm.jefferson.edu/MINTbase/	A framework for the interactive exploration of mitochondrial and nuclear tRNA fragments.	[193]
Basis	LNCipedia	lncRNA	https://lncipedia.org	A public database for lncRNA sequence and annotation.	[199]
	LNCediting	lncRNA	http://bioinfo.life.hust.edu.cn/LNCediting/	This database provides a comprehensive resource for the functional prediction of RNA editing in lncRNAs.	[200]
	lncRNAdb v2.0	lncRNA	http://lncrnadb.com/	This database provides comprehensive annotations of eukaryotic lncRNAs.	[201]
	LncRNAWiki	lncRNA	http://lncrna.big.ac.cn	This database is a publicly editable and open-content platform for community curation of human lncRNAs.	[202]
	LncBook	lncRNA	http://bigd.big.ac.cn/lncbook	This database is a curated knowledgebase of human lncRNAs.	[203]
	MONOCLdb	lncRNA	https://www.monocldb.org/	20,728 mouse lncRNA genes.	[204]
	NONCODE	lncRNA	http://www.bioinfo.org/noncode/	An interactive database that aims to present the most complete collection and annotation of ncRNAs especially lncRNAs from 17 species.	[205]
	CircAtlas	circRNA	http://circatlas.biols.ac.cn/	An integrated resource of one million highly accurate circular RNAs from 1070 vertebrate transcriptomes.	[206]
	circBase	circRNA	http://www.circbase.org/	A database containing thousands of recently identified circRNAs in eukaryotic cells.	[207]
	CIRCpedia v2	circRNA	http://www.picb.ac.cn/rnomics/circpedia	A database for comprehensive circRNA annotation from over 180 RNA-seq datasets across six different species.	[208]
	TSCD	circRNA	http://gb.whu.edu.cn/TSCD	A tissue-specific circRNA database from RNA-seq datasets and characterized the features of circRNAs in human and mouse.	[209]
	starBase v2.0	miRNA	http://starbase.sysu.edu.cn/	A database decoding miRNA-ceRNA, miRNA-ncRNA, and protein–RNA interaction networks from large-scale CLIP-Seq data.	[210]
	miRTarBase	miRNA	http://mirtarbase.cuhk.edu.cn/php/index.php	A resource for experimentally validated microRNA-target interactions.	[211]
	miRmine	miRNA	http://guanlab.ccmb.med.umich.edu/mirmine	A database of human miRNA expression profiles.	[212]
	EVmiRNA	miRNA	http://bioinfo.life.hust.edu.cn/EVmiRNA#!/	A database focusing on miRNA expression profiles in extracellular vesicles.	[213]
	miRGate	miRNA	http://mirgate.bioinfo.cnio.es/miRGate/	A curated database of human, mouse, and rat miRNA–mRNA targets.	[214]
	miRBase	miRNA	http://www.mirbase.org/	A database containing microRNA sequences from 271 organisms: 38,589 hairpin precursors and 48,860 mature microRNAs.	[215]
	DIANA-TarBase v8	miRNA	http://www.microrna.gr/tarbase	A reference database devoted to the indexing of experimentally supported miRNA targets.	[216]
	DASHR 2.0	small ncRNA	http://lisanwanglab.org/DASHR	A database that integrates human small ncRNA gene and mature products derived from all major RNA classes.	[217]

Several specific RNA-seq datasets have revealed the subcellular locations and potential interactomes of ncRNAs, which provide more real information than what is learned from bioinformatics prediction. There are some databases that provide high-quality RNA subcellular location resources in accordance with the results of subcellular compartment sequencing, such as RNALocate [218] and LncATLAS [219]. RNALocate documents more than 37,700 manually curated RNA subcellular location entries with experimental evidence, and it has data on 65 organisms, 42 subcellular locations (such as cytoplasm, nucleus, endoplasmic reticulum), and 9 RNA categories, such as lncRNAs [218]. However, thus far, few interactome database of ncRNAs except miRNA [210, 211, 216], has been established based on experimental techniques and sequencing. NPInter v3.0 is a database of ncRNA-associated interactions based on experimental techniques followed by high-throughput sequencing, such as crosslinking and immunoprecipitation followed by deep sequencing (CLIP-seq) [220], and chromatin isolation by RNA purification followed by high-throughput sequencing (ChIRP-seq) [161, 221]. NPInter v3.0 documented approximately 500,000 interactions in 188 tissues (or cell lines) from 68 kinds of experiments and predicted the functions of lncRNAs in humans on the basis of their interactions in the database [221]. Furthermore, a database of RNA interactomes identified by sequencing at the transcriptome scale is lacking, and it is needed for identification of novel functional ncRNAs.

### Application of cancer-related ncRNA identification for diagnosis

Due to their highly tissue-specific expression patterns identified by various sequencing techniques and their key roles in regulating biological activity in cancer, ncRNAs, including miRNAs, lncRNAs, and circRNAs, are generally considered to have potential as novel biomarkers for cancer diagnosis [20, 222, 223]. This section aims to present new developments in diagnostic kits for cancer diagnosis by the analysis of cancer-related ncRNAs.

Cancer seriously threatens the human life and gives rise to an enormous burden on society. However, the incidence and mortality of cancer could be decreased effectively by preventative measures, including early detection tests and monitoring of cancer prognosis. Therefore, searching for novel biomarkers that are easy to use, are not invasive, and exhibit high sensitivity, specificity, and stability for cancer diagnosis and prognosis has been a key clinical translational strategy. In addition to the features of specific expression patterns, some types of ncRNAs, such as miRNAs, lncRNAs, and circRNAs, have also been shown to be relatively stable in serum, plasma, saliva, or urine, which can be easier to collect and is less harmful or invasive for patients than other collection methods. In the past few years, seeking novel biomarkers in cancer diagnosis has mainly focused on miRNAs [224]. Recently, growing research has shown that other ncRNAs, especially lncRNAs and circRNAs, could also serve as a hallmark of carcinomas.

MiRNAs, lncRNAs, and circRNAs have been observed to have highly specific expression patterns in diverse types of cancers, and this aberrant expression usually occurs in certain tumor cells or cancer tissues at a specific stage of disease progression [13, 14, 225]. According to patent searches in resources such as the EPO (https://worldwide.espacenet.com), there are growing uses of these three types of ncRNAs in the preparation of diagnostic kits for various cancers, including hepatocellular, cervical, stomach, liver, breast, prostatic, and bladder cancers (Table 3). Generally, detecting cancer-related nucleic acids in patient samples using qRT-PCR with specific primers or probes is the main method for diagnosis based on ncRNAs, which is also the primary approach for diagnosing disease in the recent COVID-19 (CoronaVirusDisease2019) pandemic [226]. For example, a recent patent provided a circRNA hsacirc_0028185 qPCR assay kit for the diagnosis of hepatocellular carcinoma. By detecting expression changes of serum hsacirc_0028185, it is possible to assess the occurrence and development of hepatocellular carcinoma. Another sample is that lncRNA-AC006159.3 in the blood could be used for the diagnostic kit to rapidly speculate the cetuximab-resistant possibility of rectal cancer. Briefly, the lower the expression level of lncRNA-AC006159.3, the higher the possibility of cetuximab resistance. In addition, a patent provided application of miRNA-410 in preparation of a prostatic cancer diagnostic kit.

**Table 3:** Developments in diagnostic kits for cancer diagnosis (EPO https://worldwide.espacenet.com)

Species	Name	Expression in cancer	Diseases	Application	Patent number
circRNA	hsacirc_0028185	Up	Hepatocellular carcinoma	Cancer auxiliary diagnosis	CN111004850A (2020)
circRNA	hsa_circ_001477	Up	Gastric cancer	Cancer diagnosis	CN110129324A (2019)
circRNA	hsa_circRNA_012515	Up	Non-small cell lung cancer	Cancer diagnosis	CN110592223A (2019)
circRNA	hsa_circRNA_405124 or hsa_circ_0012152	Up	Leukemia	Cancer early diagnosis	CN109593859A (2019)
circRNA	circ_104075	Up	Liver cancer	Cancer diagnosis	CN109161595A (2019)
circRNA	circ3823	Up	Colorectal cancer	Cancer early diagnosis	CN110592220A (2019)
circRNA	hsa_circ_0021977	Up	Breast cancer	Cancer diagnosis	CN109022583A (2018)
circRNA	hsa_circ_0012755	Up	Prostate cancer	Cancer diagnosis	CN108624688A (2018)
circRNA	circ_0047921, circ_0007761 and circ_0056285	Up	Non-small cell lung cancer	Cancer early diagnosis	CN108179190A (2018)
circRNA	hsa-circRPL15-001	Up	Chronic lymphocytic leukemia	Cancer diagnosis	CN109055564A (2018)
circRNA	has_circ_0117909	Up	Acute lymphoblastic leukemia	Cancer diagnosis	CN107937522A (2017)
	has_circ_0005720	Down			
circRNA	cRNA-ZFR	Up	Bladder cancer	Cancer diagnosis	CN106011139A (2016)
lncRNA	lncRNA-AC006159.3	Down	Colorectal cancer	Cetuximab-resistance diagnosis	CN108949993A (2018)
lncRNA	lncRNAXLOC_004122, Linc00467 and lncRNAA1049452	Up	Breast cancer	Cancer bone metastasis diagnosis	CN107699619A (2017)
lncRNA	LncRNA GENE NO.9	Up	Bladder cancer	Cancer diagnosis	CN107267636A (2017)
lncRNA	LINC00516	Up	Lung cancer	Cancer or cancer metastasis diagnosis	CN108998528A (2018)
lncRNA	LSAMP-AS1	Up	Gastric cancer	Cancer diagnosis	CN110628915A (2019)
miRNA	miRNA-4692	Down	Hepatocellular carcinoma	Cancer diagnosis	CN107604065A (2018)
miRNA	miRNA-1266	Up	Endometrial carcinoma	Cancer diagnosis	CN105907883A (2016)
miRNA	miR-320	Down	Cervical cancer	Cancer early diagnosis	CN105506076A (2016)
miRNA	miRNA-2116	Up	Lung adenocarcinoma	Cancer metastasis diagnosis	CN104774966A (2015)
miRNA	miRNA-410	Up	Prostate cancer	Cancer diagnosis	CN104651492A (2015)
miRNA	miRNA-1262	Up	Acute myeloid leukemia	Cancer diagnosis	CN105063052A (2015)

It is noteworthy that the same RNA may be aberrantly expressed in many types of cancers, which allows the same RNA to be used to diagnose different kinds of cancers. Moreover, the RNA-seq data show that a number of diverse species of ncRNAs are dysregulated in cancer samples compared to normal tissues, suggesting that diagnostic kits can be designed to detect multiple ncRNAs at the same time for more efficient cancer diagnosis.

## Conclusion and perspective

The tissue-specific expression patterns, complicated regulatory networks, and emerging roles all suggest that ncRNAs are not simply debris or side products of transcriptional processes or aberrant splicing; rather, they are important regulatory molecules [102]. New technologies have endlessly emerged with different goals in ncRNA identification in multiple areas of research, including detection of ncRNA expression at the transcriptome scale, identification of novel ncRNA categories, searching for potential functional RNA within specific subcellular compartments, or discovering applicable biomarkers for cancer diagnosis. There are also some ncRNA-associated databases that provide multiple ncRNA information to enable further functional RNA investigations. Moreover, with the increasing number of studies on cancer-associated ncRNAs, translational applications of specific ncRNA identification for clinical diagnosis have been developed, such as diagnostic kits.

It is noteworthy that different RNA categories can be generated from the same regions of DNA, and they can share the same sequences. In addition to small ncRNAs derived from snoRNAs, tRNAs, or rRNAs, which can play a role in the miRNA-like pathway, some long nonpolyadenylated transcripts, such as sno-lncRNAs and circRNAs, have also been found to be generated from the genetic sequences of well-known ncRNAs [10, 37, 42, 126, 139]. Sno-lncRNAs have the same classical stem-loop as snoRNAs originating from the same genomic regions. However, both types of ncRNAs have been verified to have individual functions and to be important regulatory molecules in biological processes [42]. Another example is circANRIL, a circRNA formed from the lncRNA ANRIL, which performs functions in apoptosis and proliferation that are the opposite of the functions of ANRIL [227]. Taken together, these results suggest that transcripts derived from canonical DNA regions have functions in addition to their classical ones by interacting with nonclassical binding molecules or by being located in novel components, which indicates that the transcriptome extends far beyond the genome. A larger RNA world is waiting for us to explore.

High-throughput sequencing with purposeful sample preparation not only has uncovered novel species of ncRNAs but also has mapped the interaction networks and subcellular locations of ncRNAs [221]. In accordance with sequencing data of the RNA-associated interactome and RNA subcellular locations that have been determined at the transcriptome scale, a series of noncoding transcripts, especially lncRNAs and circRNAs, exhibit specific distributions in organelles, protein complexes, or subcellular structures [228]. These results further indicate that ncRNAs are functional molecules playing roles in specific compartments, providing a pool of candidates for us to search for specifically functional ncRNAs. Using these sequencing methods, a huge number of snoRNAs have been found to be enriched on chromatin, robustly suggesting that other potential functions of these well-known small ncRNAs are located in a nonclassical compartment [71]. However, the functions of chromatin-associated snoRNAs remain unanswered. In addition, thousands of potential functional ncRNAs with specific interactions or locations have been discovered and await further investigation.

Due to the features of specific expression patterns in cancers and relatively high stability in serum, plasma, saliva, or urine, ncRNAs especially miRNAs, lncRNAs, and circRNAs are generally considered to have potential as non-invasive diagnostic biomarkers for cancers. A growing number of researehes have provided the suitable ncRNA candidates for diagnosis of different cancers and increasing patents about preparation of ncRNA diagnostic kits for cancer diagnosis have been approved. However, most of these candidate ncRNAs are still in the preclinical stages. In addition, the results of some studies evaluating the potential of ncRNAs as biomarkers are conflicting [20]. Thus, more accurate evaluation of RNA expression pattern in larger cohorts of clinical data are needed to reconcile the controversies.

## Data Availability

The material supporting the conclusion of this study has been included within the article.

## Abbreviations

ncRNA: Noncoding RNA
lncRNA: Long noncoding RNA
circRNA: Circular RNA
mRNA: Message RNA
H3K4me3: Trimethylation of lysine 4 of histone 3
H3K36me3: Trimethylation of lysine 36 of histone 3
lincRNA: Long intergenic ncRNA
H3K4m1: Monomethylation of lysine 4 of histone 3
H3K27ac: Histone H3 acetylation at lysine 27
eRNA: Enhancer-derived RNA
3D: 3 Dimensions
pre-RNA: Precursor RNA
snoRNA: Small nucleolar RNA
rRNA: Ribosomal RNA
tRNA: Transfer RNA
sno-lncRNA: SnoRNA-related lncRNA
rRF: Ribosomal RNA-derived fragment
tsRNA: tRNA-derived small RNA
sdRNA: snoRNA-derived RNA
ORF: Open reading frame
SRA: Steroid receptor RNA activator
Xist: X inactive specific transcript
lnx3: Ligand of numb-protein X 3
Pol II: RNA polymerase II
snoRBP: snoRNA binding protein
RNA-seq: RNA sequencing
XPO1: Exportin 1
CAMSAP1: Calmodulin-regulated spectrin-associated protein 1
EIF3J: Eukaryotic translation initiation factor 3 subunit J
PAIP2: Poly(A)-binding protein-interacting protein 2
miRNA: MicroRNA
siRNA: Small interfering RNA
piRNA: Piwi-interacting RNA
Ago: Argonaute
RISCs: RNA-induced silencing complexes
RBP: RNA-binding protein
vRNA: Vault RNA
VTRNA: Vault RNA gene
Pol III: RNA polymerase III
Dicer: Ribonuclease III
cDNA: Complementary DNA
HOTAIR: HOX antisense intergenic RNA
NGS: Next-generation sequencing
PRC2: Polycomb repressive complex
scRNA-seq: Single-cell RNA sequencing
FACS: Fluorescence-activated cell sorting
DP-seq: Designed primer-based sequencing
MMLV RT: Moloney murine leukemia virus reverse transcriptase
PCR: Polymerase chain reaction
SUPeR-seq: Single-cell universal poly(A)-independent sequencing
RamDA-seq: Random displacement amplification sequencing
RT-RamDA: RT with random displacement amplification
NSR: Not-so-random primer
Neat1: Nuclear paraspeckle assembly transcript 1
GRO-seq: Global run-on sequencing
RT: Reverse transcription
GRID-seq: Global RNA interactions with DNA by deep sequencing
DSG: Disuccinimidyl glutarate
ssRNA: Single-stranded RNA
dsDNA: A double-stranded DNA
ChAR-seq: Chromatin-associated RNA sequencing
caRNA: Chromatin-associated RNA
TSSs: Transcription star sites
snRNA: Small nuclear RNA
CLASH: Cross-linking ligation and sequencing of hybrids
RIPPLiT: RNA immunoprecipitation and proximity ligation in tandem
MARIO: Mapping the RNA interactome in vivo
PARIS: Psoralen analysis of RNA interactions and structures
LIGR-seq: Ligation of interacting RNA followed by high-throughput sequencing
SPLASH: Sequencing of psoralen cross-linked, ligated, and selected hybrids
RIC: RNA in situ conformation sequencing
ISH: In situ hybridization
smFISH: Single-molecule fluorescence ISH
FISSEQ: Fluorescent in situ RNA sequencing
STARmap: Spatially resolved transcript amplicon readout mapping
CeFra-seq: Biochemical cell fractionation combined with RNA-seq
APEX: Engineered ascorbate peroxidase
RIP: RNA immunoprecipitation
CLIP-seq: Immunoprecipitation followed by deep sequencing
ChIRP-seq: Chromatin isolation by RNA purification followed by high-throughput sequencing
